# Anti-cancer effects of plant extracts in head and neck squamous cell carcinoma

**DOI:** 10.3389/fphar.2026.1889422

**Published:** 2026-08-24

**Authors:** Haoran Zhou, Yafei Li, Lu Liu, Bo Li

**Affiliations:** Department of Oral Anatomy and Physiology, Jilin Provincial Key Laboratory of Oral Biomedical Engineering, Hospital of Stomatology, Jilin University, Changchun, China

**Keywords:** anti-cancer, HNSCC, mechanism, plant extracts, therapy

## Abstract

Current treatments for head and neck squamous cell carcinoma (HNSCC) often encounter difficulties such as local tumor recurrence, distant metastasis, drug resistance, and significant toxic side effects. Plant extracts have become important resources for the development of anti-HNSCC drugs due to their multi-target action mechanisms, abundance of bioactive components, reversal of drug resistance, and high selectivity/low toxicity. The challenges faced by plant extracts during anti-cancer clinical translation include low bioavailability, unclear mechanisms of action, complex components, and difficulties in standardization. This review systematically summarizes the latest research progress on the anti-cancer effects of plant extracts in HNSCC and their regulatory signaling pathways. Various plant extracts, such as resveratrol, curcumin, quercetin, and anthocyanins, have anti-HNSCC effects, including inducing apoptosis, inhibiting proliferation, blocking metastasis, and reversing chemoresistance. The key underlying molecular events are primarily mediated by the PI3K/AKT/mTOR, MAPK, and JAK/STAT3 cascades. Future research directions for plant extracts in combating HNSCC include developing intelligent nano-drug delivery systems, integrating multi-omics with artificial intelligence, establishing innovative research models, and regulating the tumor microenvironment.

## Graphical Abstract

**Figure:**
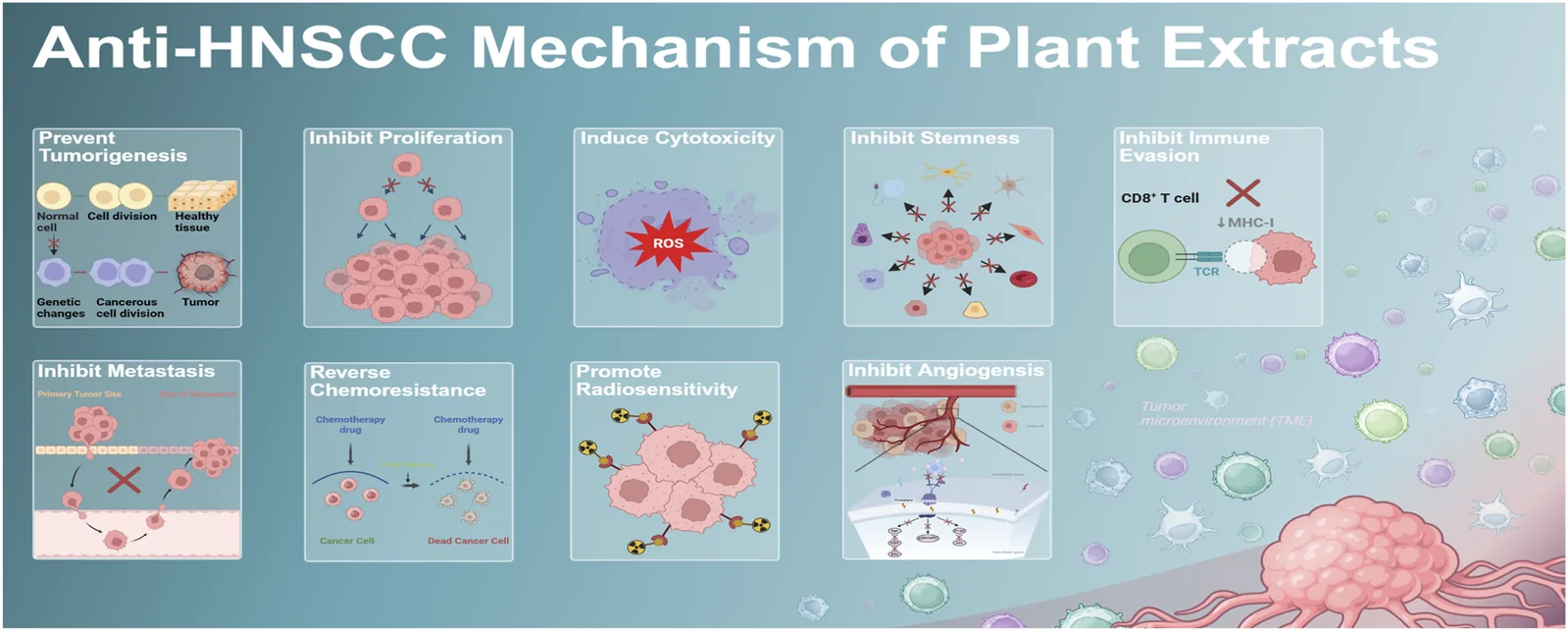
Mechanisms of plant extracts against HNSCC. These extracts exert antitumor effects through multiple mechanisms, including prevention of tumorigenesis, induction of cytotoxicity, and inhibition of cellular proliferation, stemness, metastasis, angiogenesis, and immune evasion. Additionally, chemoresistance is reversed and radiosensitivity is enhanced.

## 1 Introduction

Head and neck squamous cell carcinoma (HNSCC) represents the sixth most common cancer worldwide, accounting for approximately 550,000 new cases each year and a 5 year survival rate of less than 50% (Vitale et al., 2026). It is characterized by early high incidence of lymph node metastasis, the synergy of epithelial mesenchymal transition (EMT) and tumor stem cell-driven invasion and resistance, as well as multiple treatment resistances mediated by the immunosuppressive microenvironment (Li C. et al., 2024). The treatment approaches for HNSCC include surgery, radiotherapy, chemotherapy, anti-EGFR targeted therapy (cetuximab), and immunotherapy represented by PD-1 inhibitors (pembrolizumab, nivolumab) (Cao et al., 2024). Current treatments often encounter difficulties such as tumor recurrence, metastasis, drug resistance, and significant toxic side effects (Liu L. et al., 2025; Liu X. et al., 2025; Mei et al., 2020; Solomon et al., 2018). There is a pressing need to develop novel adjuvant or alternative therapeutic strategies for HNSCC to overcome this therapeutic predicament.

**FIGURE 1:**
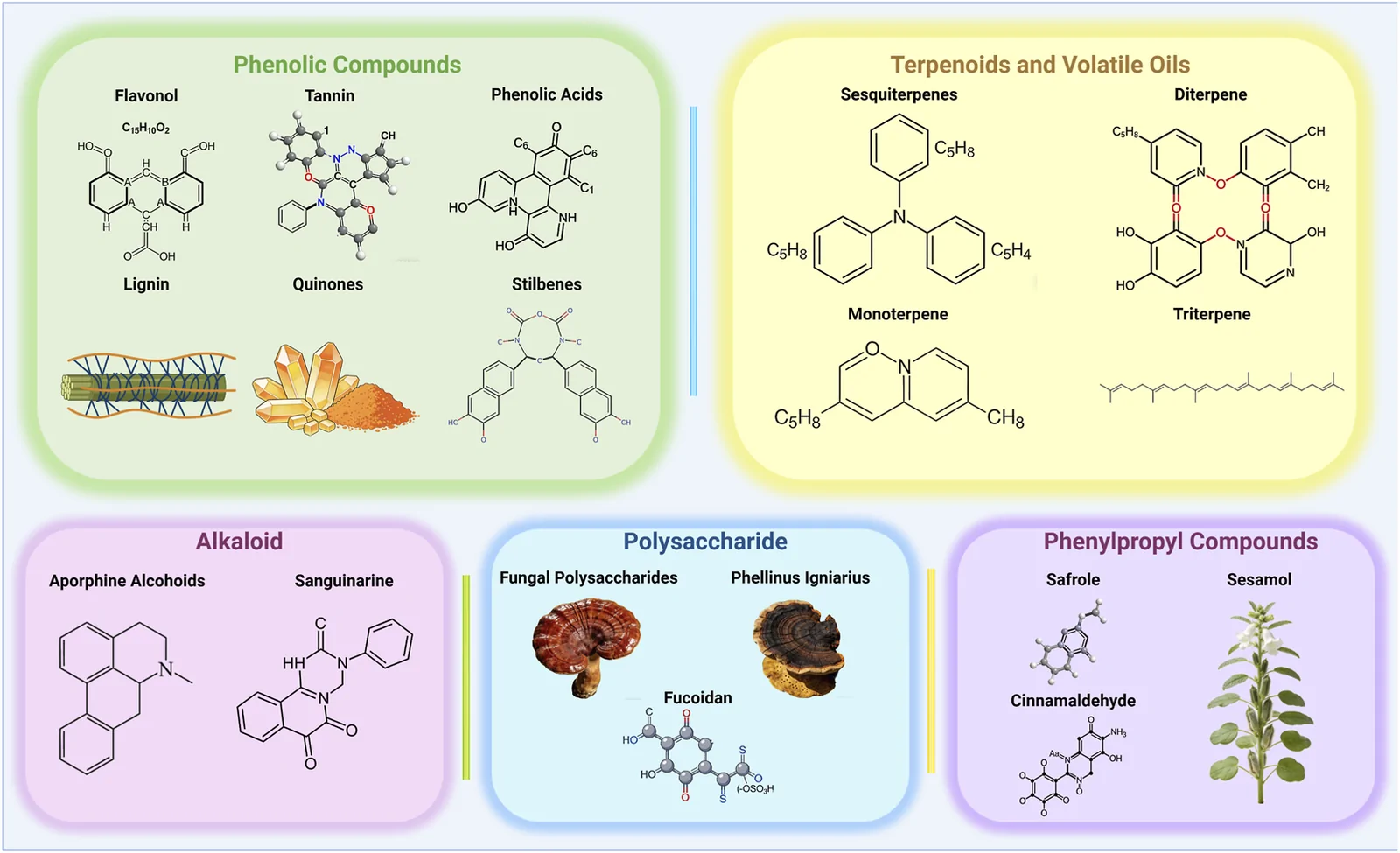
Classification of plant extracts with anti-HNSCC activity. Bioactive plant extracts are categorized into the following classes: phenolic compounds, terpenoids and volatile oils, alkaloids, polysaccharides, and phenylpropanoids.

The characteristics of plant extracts and their derivatives include structural diversity, diverse biological activities, low toxicity and side effects, and wide range of sources (Xing et al., 2023). Researchers are increasingly interested in discovering novel plant extract-derived potential regulators for anti-tumor treatment (Ansari et al., 2017; Bańkosz and Tyliszczak, 2024; Park et al., 2020; Song et al., 2023; Huang et al., 2021; Zhou et al., 2024). Some well-known plant extracts, including polyphenols (e.g., curcumin and resveratrol), terpenoids (e.g., paclitaxel and artesunate), polysaccharides (e.g., fungal polysaccharides and Ganoderma lucidum polysaccharides), and saponins, have been proven to exhibit anti-tumor effects (Deng et al., 2020; Hołota and Posmyk, 2025; Fan et al., 2024; Jiang et al., 2022). Plant extracts exhibit anti-tumor activity through multiple mechanisms, including induction of apoptosis, inhibition of cellular proliferation, suppression of angiogenesis, and attenuation of invasion and migration, while concurrently protecting normal cells from the cytotoxic effects associated with conventional chemotherapy and radiotherapy (Li et al., 2025). Plant extracts have become important resources for the development of anti-HNSCC drugs due to their multi-target action mechanisms, abundance of bioactive components, reversal of drug resistance, and high selectivity/low toxicity (Figure 1).

The challenges faced by plant extracts during clinical translation include low bioavailability, unclear mechanisms of action, complex components, and difficulties in standardization (Ren et al., 2021). This review systematically summarizes the latest research progress on the anti-cancer effects of plant extracts on HNSCC and their regulatory signaling pathways. To ensure a comprehensive and reproducible coverage of the literature, we systematically searched the Web of Science Core Collection for English-language, peer-reviewed articles published between 2021 and 2025. The search strategy employed the following keywords: “plant extract,” “HNSCC,” “head and neck squamous cell carcinoma,” “OSCC,” “oral squamous cell carcinoma.” Original research articles that demonstrated anti-cancer effects against HNSCC were designated as the core literature. Reviews, conference abstracts, editorials, commentaries, and non-English publications were excluded. To avoid ambiguity, this review will differentiate between three types of botanical interventions: whole/crude extracts, enriched fractions, and isolated pure compounds.

## 2 Anti-HNSCC effects of plant extracts

### 2.1 Phenolic compound

#### 2.1.1 Flavonoids

**FIGURE 2:**
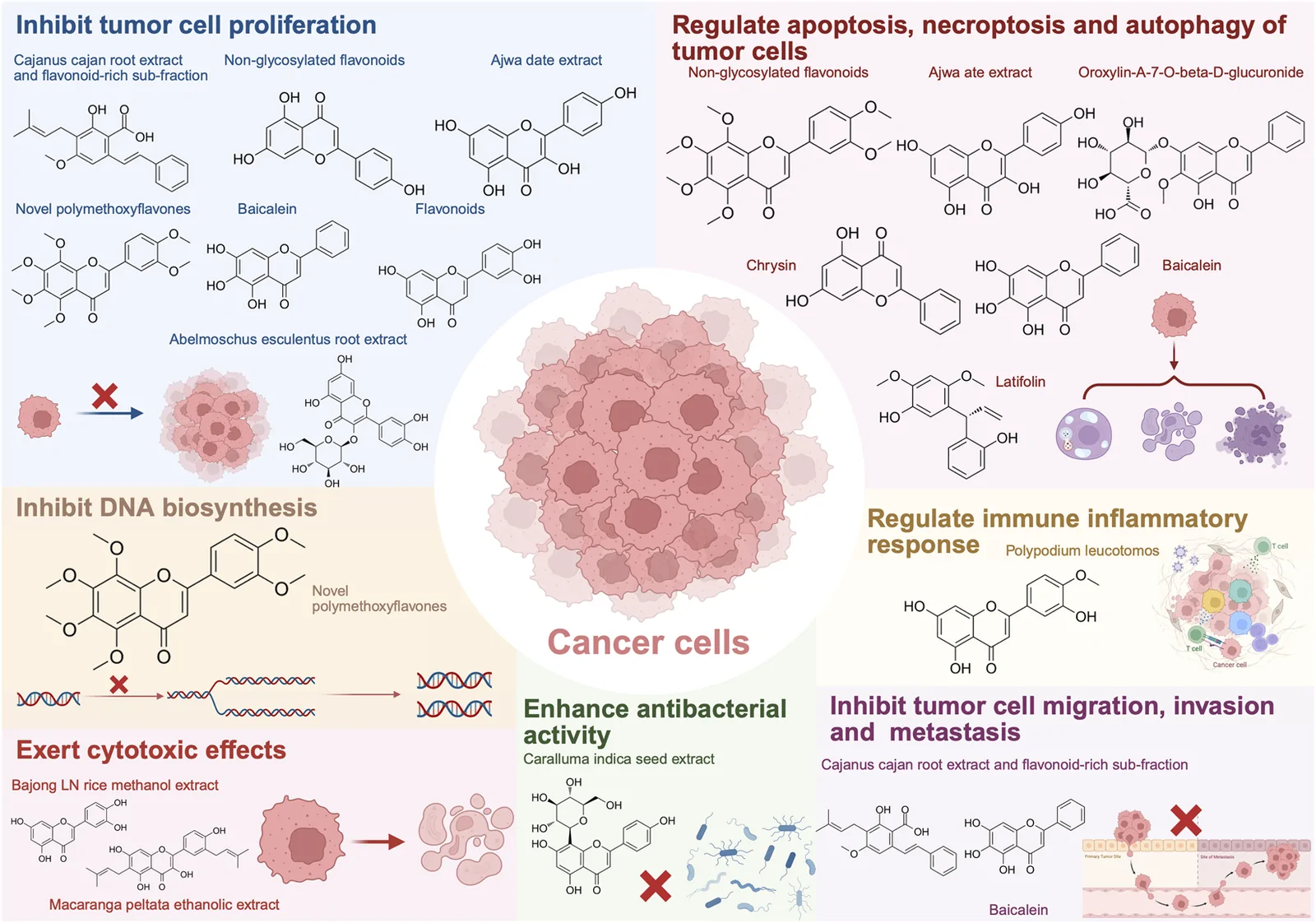
Anti-tumor mechanisms of flavonoid-based plant extracts in HNSCC. The key activities of these compounds comprise: inhibition of tumor cell proliferation, migration, invasion, and metastasis; regulation of apoptosis, necroptosis, and autophagy; suppression of DNA biosynthesis; exertion of direct cytotoxic effects; modulation of immune-inflammatory responses; and enhancement of antimicrobial activity.

Flavonoids, including anthocyanins, flavones, flavonols and isoflavones, play critical roles in inhibiting the proliferation, migration and gene expression of HNSCC cells and inducing apoptosis and cytotoxicity responses, thereby restraining the progression of oral squamous cell carcinoma (OSCC) and diminishing its risk (Figure 2).

##### 2.1.1.1 Anthocyanidin

Bilberry powder extract (enriched in anthocyanins) restrains the migratory, invasive, proliferative, and tumorigenic capacities of OSCC (Mauramo et al., 2021). The isolated anthocyanins suppress OSCC development in oral cells via the inhibition of DNA adducts induced by benzo[a]pyrene (Chen K-M. et al., 2024). In a carcinogen-induced model, the standardized black raspberry extract (enriched in anthocyanins) suppresses early-stage OSCC development through modulating the gut and oral microbiomes (Zhao et al., 2025). The isolated anthocyanin inhibits oral carcinogenesis through upregulating hydroxysteroid 11-beta dehydrogenase 2 (HSD11B2) to restore glucocorticoid balance (Nedungadi et al., 2022) (Table 1).

**TABLE 1:** The anti-tumor effects and mechanisms of phenolic compound against HNSCC.

Classification	Plant extracts	Mechanism of action	Tumor progression	Tumor	Ref
Anthocyanidin	Bilberry powder extract (enriched in anthocyanins)	NA	Inhibit migration, invasion, proliferation and tumor growth	OSCC	Mauramo et al. (2021)
	The isolated anthocyanins	Inhibits benzo[a]pyrene-induced DNA adducts	Restrains OSCC development	OSCC	Chen K.-M. et al. (2024)
	The standardized black raspberry extract (enriched in anthocyanins)	Modulates gut and oral microbiomes in a carcinogen-induced model	Suppresses early-stage OSCC development	OSCC	Zhao et al. (2025)
	The isolated anthocyanin	Upregulates HSD11B2 to restore glucocorticoid balance	Inhibits oral carcinogenesis	OSCC	Nedungadi et al. (2022)
Flavones	BLN-ME	NA	Exerts cytotoxic effect	OSCC	Ahmad et al. (2024)
	*Macaranga peltata* ethanolic extract	NA	Exerts cytotoxic and genotoxic effects	OSCC	Nizar et al. (2024)
	*Abelmoschus esculentus* root extract	NA	Inhibits proliferation	OSCC	Desai et al. (2025)
	The standardized Cajanus cajan (L.) Millsp. root extract (enriched in flavones)	NA	Inhibits proliferation and metastasis	OSCC	Vo et al. (2022)
	The isolated non-glycosylated flavonoids	NA	Inhibits proliferation and induces apoptosis	OSCC	de Queiroz et al. (2023)
	Ajwa date extract	NA	Inhibits proliferation and induces apoptosis	OSCC	Shahbaz et al. (2022)
	The isolated latif	Inhibits PI3K/AKT/mTOR/p70S6K signaling pathway	Induces apoptosis, inhibits cell survival, proliferation and necroptosis	OSCC	Yun et al. (2022)
	The isolated chrysin	Oxidative DNA damage	Promotes apoptosis	OSCC	Chen K.-M. et al. (2023)
	The isolated oroxylin-A-7-O-beta-D-glucuronide	Disrupts mitochondrial microenvironment	Induces mitochondrial-dependent apoptosis	OSCC	Kameyanda Poonacha et al. (2021)
	*Polypodium leucotomos* extract	Modulates immune-inflammatory responses	Suppresses TSCC carcinogenesis and tumor progression	TSCC	Lacerda et al. (2023)
	*Caralluma indica* seed extract	Antimicrobial activity and biofilm suppression	Reduces oral cancer progression risk	Oral cancer	Ramalingam et al. (2024)
	The isolated novel polymethoxyflavones from *Hottonia palustris* evoke	DNA replication blockade	Inhibit DNA biosynthesis and suppress proliferation	OSCC	Strawa et al. (2022)
	The isolated baicalein	Suppresses PI3K-AKT signaling	Inhibits invasion, migration, and proliferation, and promotes cell apoptosis	OSCC	Hou et al. (2021)
	A standardized PME (enriched in flavonoids)	Arresting cell cycle and positively regulating p53, HIF-1α and CDH1	Inhibits proliferation	OSCC	Fonseca-Benitez et al. (2022)
Flavonol	Root and fruit extracts of *Withania somnifera* (L.)	NA	Demonstrate cytotoxicity	OSCC	Al Awadh et al. (2024)
	The isolated quercetin	NA	Exerts cytotoxicity	OSCC	Assef et al. (2026)
	The isolated quercetin and rutin	Modulates tumor suppressor genes	Suppress proliferation	OSCC	Sharma et al. (2023)
Isoflavones	The isolated biochanin A	Enhances carcinogen detoxification and antioxidant defenses	Prevents OSCC development	OSCC	Nivedha et al. (2024)
	The isolated SFB	Inhibits MAPK (ERK1/2, p38 and JNK1/2) signaling pathways	Promotes apoptosis	HNSCC	Hsieh et al. (2023)
Tannin	The isolated gallotannins	NA	Induces cell apoptosis, and inhibits proliferation and migration	OSCC	Devi et al. (2025)
	*Agrimonia pilosa* Ledeb. root extract	Reduces expression of proteins in hnRNP family	Exhibits antitumor effects	OSCC	Kim T.-Y. et al. (2022)
	The isolated polymeric black tea polyphenols	Down-regulating EGFR/AKT/mTOR signaling pathway	Inhibit proliferation, apoptosis and angiogenesis	oral cancers	Nimbalkar et al. (2022)
Lignin	The isolated lignans cubebin and methylcubebin	NA	Have anti-proliferative, anti-migration and genotoxic effects	HNSCC	Gusson-Zanetoni et al. (2022)
	The isolated machilin D	Suppresses adhesion molecules (including FAK/Src pathway), inhibits PI3K/AKT/mTOR/p70S6K pathway and MAPKs, and increasing LC3I/II and Beclin1 and reducing p62	Exhibits significant cytotoxicity against OSCC cells, inhibits cell adhesion, migration, necroptosis and invasion, leads to cell apoptosis and promotes autophagy	OSCC	Yun et al. (2023)
	The isolated pinoresinol	EMT marker modulation	Inhibits OSCC cells proliferation, induces apoptosis and suppresses migration	OSCC	Bai et al. (2023)
	The isolated schisandrin A	Targets PI3K/AKT/GSK3β signaling pathway	Inhibits the progression of HNSCC	HNSCC	Wu et al. (2026)
Phenolic acids	Bioadhesive films delivering *Usnea barbata* extract	NA	Trigger apoptosis of oral cancer cells and inhibit DNA synthesis	OSCC	Popovici et al. (2022a)
	Perilla phenols(enriched in caffeic acid and rosmarinic acid)	Reverse chemoresistance and enhancing EGFR-targeted therapy	Induce OSCC cells apoptosis	OSCC	Lee et al. (2023)
	The isolated GAME and PGG	Halt G2 phase and suppress AKT phosphorylation	Inhibit cell proliferation	OSCC	Nakamura et al. (2021)
	The isolated methyl gallic acid	Improves bioavailability and drug release kinetics significantly	Enhances cytotoxicity	OSCC	Rajan et al. (2023)
	*Usnea barbata* oil extract	Synergistic action of lichen metabolites and canola oil phytoconstituents	Induces DNA damage and pro-death autophagy	OSCC	Popovici et al. (2022b)
	*Usnea barbata* extract-loaded mucoadhesive films	NA	Inhibits OSCC proliferation and induce apoptosis and autophagy	OSCC	Popovici et al. (2022c)
Quinone	Fermented wheat germ extract	NA	Suppresses migration, invasion and OTSCC cell viability	OTSCC	Zh et al. (2022)
	The isolated hypericin	NA	Inhibits TSCC proliferation and induces apoptosis	TSCC	Tondro et al. (2024)
	The isolated CE and AE	NA	Have significant apoptotic and anti-migration potentials	OSCC	Shams et al. (2023)
​	The isolated PLB	Activates JNK and inhibits AKT/mTOR pathway	Triggers apoptosis and protective autophagy, inhibits growth of TSCC	TSCC	Xue et al. (2021)
	The isolated PLB	NA	Inhibits growth, proliferation and induces apoptosis of drug-resistant	OSCC	Lin et al. (2023)
Stilbenes	*Pterocarpus marsupium* bark extract	NA	Exhibits anti-cancer and antioxidant effects	OSCC	Samal et al. (2024)
	The isolated scirpusin B	Suppresses cyclin D1	Inhibits OSCC proliferation	OSCC	Purohit et al. (2024)
	​	Survivin downregulation	Reverses apoptosis resistance	OSCC	Purohit et al. (2024)
	​	VEGF-A suppression	Blocks angiogenesis	OSCC	Purohit et al. (2024)
	​	Targets TNFα/COX-2	Reduces inflammation	OSCC	Purohit et al. (2024)
Curcuminoids	The isolated curcumin	NA	Affects cell proliferation, apoptosis, and metastasis	HNSCC	Li et al. (2026)

##### 2.1.1.2 Flavones

Flavonoid compounds restrain the genesis and evolution of HNSCC. They can do so by inducing apoptosis and autophagy, inhibiting proliferation, regulating immune inflammatory responses, exerting cytotoxic effects, and suppressing migration. Bajong LN Rice Methanol Extract has been shown to have a cytotoxic effect specifically against human squamous cell carcinoma (Ahmad et al., 2024). *Macaranga peltata* ethanolic extract has been shown to exert cytotoxic and genotoxic effects on OSCC cells (Nizar et al., 2024). *Abelmoschus esculentus* root extract inhibits OSCC proliferation (Desai et al., 2025). The standardized Cajanus cajan (L.) Millsp. root extract (enriched in flavones) inhibits OSCC cell proliferation and metastasis (Vo et al., 2022). The isolated non-glycosylated flavonoids selectively inhibit OSCC proliferation and induce apoptosis (de Queiroz et al., 2023). Ajwa date extract inhibits OSCC cell proliferation and induces apoptosis in OSCC cells (Shahbaz et al., 2022). The isolated latif induces apoptosis by inhibiting the activity of the PI3K/AKT/mTOR/p70S6K signaling pathway, thereby inhibiting the survival and proliferation of YD-10B OSCCs, suppresses necroptosis through the dephosphorylation of necroptotic control proteins (RIP1, RIP3 and MLKL), and simultaneously inhibits autophagy-related proteins and autophagosome formation (Yun et al., 2022). The isolated chrysin promotes cisplatin-induced apoptosis through oxidative DNA damage in OSCC cells (Chen J. et al., 2023). The isolated oroxylin-A-7-O-beta-D-glucuronide induces mitochondrial-dependent apoptosis in OSCC cells by disrupting the mitochondrial microenvironment (Kameyanda Poonacha et al., 2021). *Polypodium leucotomos* extract suppresses oral tongue squamous cell carcinoma (OTSCC) carcinogenesis and tumor progression by modulating immune-inflammatory responses (Lacerda et al., 2023). *Caralluma indica* seed extract reduces oral cancer progression risk through antimicrobial activity and biofilm suppression (Ramalingam et al., 2024). The isolated novel polymethoxyflavones from *Hottonia palustris* evoke inhibit DNA biosynthesis in OSCC cells and suppress proliferation through blocking DNA replication (Strawa et al., 2022). The isolated baicalein inhibits invasion and migration and promotes apoptosis by suppressing the PI3K-AKT signaling pathway and inhibiting proliferation in OSCC cells (Hou et al., 2021). A standardized *Passiflora mollissima (Kunth) L.H. Bailey* seeds extract (PME) (enriched in flavonoids) inhibits the proliferation of OSCC cells by arresting the cell cycle and positively regulating p53, HIF-1α and CDH1 expression (Fonseca-Benitez et al., 2022). Flavonoids can inhibit the PI3K-AKT signaling pathway. These experimental studies provide a large number of theoretical bases for the clinical treatment of HNSCC with flavonoids. However, further clinical research is still needed to determine whether flavonoids can potentially transform into promising anti-tumor drug for clinical use.

##### 2.1.1.3 Flavonol

Flavonol can inhibit tumor progression through cytotoxic effects and by suppressing tumor proliferation. Root and fruit extracts of *Withania somnifera* (L.) are cytotoxic to OSCC cells (Al Awadh et al., 2024). The isolated quercetin is cytotoxic to OSCC cells (Assef et al., 2026). The isolated quercetin and rutin suppress OSCC proliferation through the modulation of tumor suppressor genes (Sharma et al., 2023). Flavonols are targeted anti-tumor drugs with potential clinical translational significance.

##### 2.1.1.4 Isoflavones

The isolated biochanin A prevents OSCC development by enhancing carcinogen detoxification and antioxidant defenses (Nivedha et al., 2024). The isolated semilicoisoflavone B (SFB) promotes the apoptosis of HNSCC cells through inhibiting MAPK (ERK1/2, p38 and JNK1/2) signaling pathways (Hsieh et al., 2023).

Overall, flavonoids can prevent the occurrence and progression of HNSCC through multiple mechanisms. First, they can inhibit the occurrence and development of OSCC by regulating the immune-inflammatory response. They can also inhibit proliferation by exerting cytotoxic effects, regulating tumor suppressor genes, and inhibiting DNA replication. They can promote cisplatin resistance through oxidative DNA damage, disrupt the mitochondrial microenvironment, increase reactive oxygen species (ROS) levels, inhibit the MAPK pathway, and downregulate survivin to induce apoptosis. They can inhibit the invasion and metastasis of HNSCC through inhibiting the expression of metastasis-related genes, the PI3K-AKT signaling pathway and regulating 141 other pathways. In addition, flavonoids can also inhibit the progression of HNSCC through interactions with the oral environment and the whole body. For example, they can reduce the risk of oral cancer progression through antibacterial activity and the inhibition of biofilm formation, restore glucocorticoid balance by upregulating HSD11B2, and regulate the intestinal and oral microbiota in carcinogen-induced models to inhibit the occurrence of oral cancer. Among all flavonoid extracts, anthocyanins are currently the focus of research on the anti-HNSCC effects of plant extracts. They can inhibit the DNA adducts induced by benzo[a]pyrene, regulate the intestinal and oral microbiota in carcinogen-induced models, and upregulate HSD11B2 to restore glucocorticoid balance, thereby playing a role in the progression of HNSCC. Further research on anthocyanins can be conducted to identify effective drugs for clinical anti-HNSCC treatment.

#### 2.1.2 Tannin

The isolated gallotannins trigger apoptosis and attenuate the proliferation and migratory capacity of SCC9 cells (Devi et al., 2025). *Agrimonia pilosa* Ledeb. root extract exerts antitumor effects on OSCC cells by reducing the expression of proteins in the hnRNP family (Kim T.-Y. et al., 2022). The isolated polymeric black tea polyphenols exert anti-proliferative and anti-angiogenic effects, as well as pro-apoptotic effects, via downregulation of the EGFR/AKT/mTOR cascade (Nimbalkar et al., 2022).

#### 2.1.3 Lignin

The isolated lignans cubebin and methylcubebin have anti-proliferative, anti-migration and genotoxic effects on HNSCC (Gusson-Zanetoni et al., 2022). By suppressing adhesion molecules (including the FAK/Src pathway), the isolated machilin D significantly inhibits OSCC cells; inhibits cell adhesion, migration, necroptosis and invasion; and leads to apoptosis through the inhibition of the PI3K/AKT/mTOR/p70S6K pathway and MAPK signaling, and it promotes autophagy by increasing LC3-I/II and Beclin1 expression and reducing p62 expression (Yun et al., 2023). The isolated pinoresinol, a lignan isolated from Ficus septica bark, inhibits OSCC cell proliferation, induces apoptosis and suppresses migration via the modulation of EMT markers (Bai et al., 2023). The isolated schisandrin A, a compound derived from the fruit of Schisandra chinensis, has been shown to inhibit the progression of HNSCC by targeting the PI3K/AKT/GSK3β signaling pathway (Wu et al., 2026).

#### 2.1.4 Phenolic acids

Phenolic acids play a role in inhibiting the progression of HNSCC by inhibiting proliferation, inducing apoptosis and exerting cytotoxic effects. Bioadhesive films delivering *Usnea barbata* extract trigger oral cancer apoptosis and inhibit DNA synthesis (Popovici et al., 2022a). Perilla phenols (enriched in caffeic acid and rosmarinic acid) induce OSCC apoptosis through reversing chemoresistance and enhancing EGFR-targeted therapy (Lee et al., 2023). The isolated Gallic acid methyl ester (GAME) and pentagalloyl glucose (PGG) inhibit cell proliferation by halting G2 phase and suppressing AKT phosphorylation (Nakamura et al., 2021). The isolated methyl gallic acid encapsulated in ethosomal nanovesicles significantly enhances the cytotoxicity against OSCC cells by improving bioavailability and drug release kinetics (Rajan et al., 2023). *Usnea barbata* oil extract induces DNA damage and pro-death autophagy in OSCC cells through the synergistic action of lichen metabolites and canola oil phytoconstituents (Popovici et al., 2022b). *Usnea barbata* extract-loaded mucoadhesive films inhibits OSCC proliferation and induces apoptosis and autophagy (Popovici et al., 2022c). In summary, phenolic acid plant extracts can first inhibit OSCC proliferation by regulating the cell cycle and can promote apoptosis and autophagy in OSCC cells through oxidative stress and DNA damage. They can also weaken the resistance of OSCC to chemoresistance and increase its toxicity to OSCC cells. In addition to single drug use, phenolic acid compounds can also have synergistic anti-HNSCC effects when combined with other compounds. However, research on phenolic acid extracts in anti-HNSCC is limited, and further studies are needed.

#### 2.1.5 Quinones

Quinone compounds inhibit the occurrence and development of OSCC by decreasing the viability of OSCC cells, inhibiting proliferation, and inducing apoptosis. Fermented wheat germ extract suppresses migration, invasion and OTSCC cell viability (Zh et al., 2022). The isolated hypericin inhibits TSCC proliferation and induces apoptosis (Tondro et al., 2024). The isolated costunolide (CE) and aloe emodin (AE) have significant apoptotic and anti-migration potentials on OSCC cells (Shams et al., 2023). The isolated plumbagin (PLB) triggers apoptosis and autophagy by activating JNK and inhibiting the AKT/mTOR pathway, thereby inhibiting the growth of TSCC (Xue et al., 2021). The isolated PLB inhibits the growth and proliferation and induces the apoptosis of drug-resistant OSCC cells (Lin et al., 2023). Overall, quinone compounds can promote apoptosis by suppressing tumor cell proliferation, inducing ROS-mediated ER stress and mitochondrial dysfunction, and inhibiting migration and cell viability, thereby preventing the occurrence and development of HNSCC. However, relatively little research has investigated the mechanism of quinone compounds in anti-HNSCC, and further exploration of its anti-HNSCC mechanism is needed.

#### 2.1.6 Stilbene

*Pterocarpus marsupium* bark extract exhibits anti-cancer and antioxidant effects against OSCC (Samal et al., 2024). The isolated scirpusin B inhibits OSCC proliferation by suppressing cyclin D1 expression, reverses apoptosis resistance via survivin downregulation, blocks angiogenesis through the suppression of VEGF-A expression, and reduces inflammation through the targeting of TNFα/COX-2 (Purohit et al., 2024).

#### 2.1.7 Curcuminoids

The isolated curcumin has been shown to suppress the tumorigenesis of HNSCC by directly targeting the transcription factors FOSL1 and JUN. This suppression occurs through various molecular mechanisms, suggesting that curcumin could serve as a potential therapeutic agent for HNSCC. The mechanisms include modulation of signaling pathways that affect cell proliferation, apoptosis, and metastasis, thereby highlighting the importance of FOSL1 and JUN as critical targets in the treatment and prevention of HNSCC (Li et al., 2026).

### 2.2 Terpenoids and volatile oils

#### 2.2.1 Monoterpenes

**FIGURE 3:**
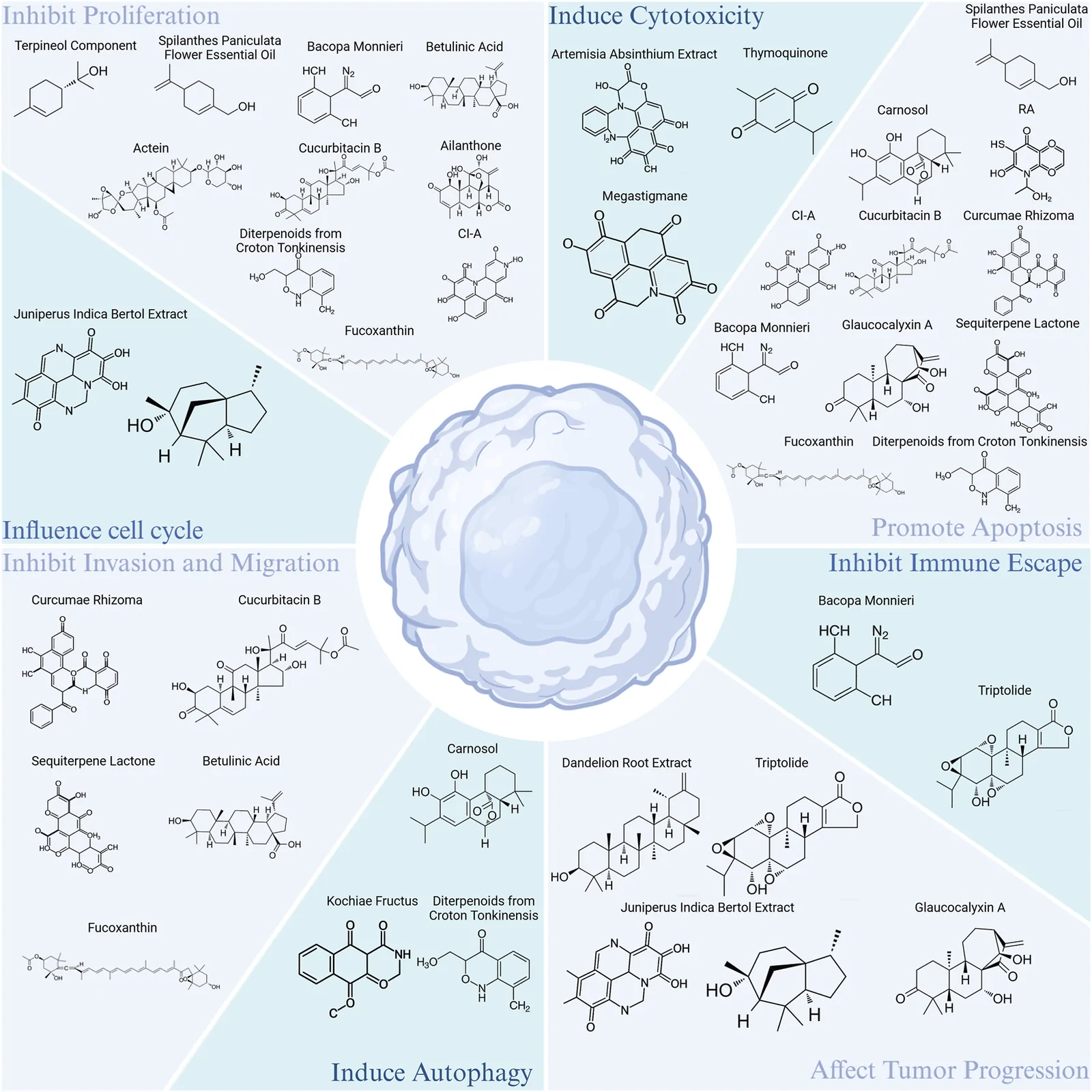
Mechanisms of action of terpenoid and volatile oils compounds against HNSCC. HNSCC progression is inhibited by terpenoids through multiple mechanisms. Specifically, proliferation, invasion, migration, and immune escape are suppressed. Furthermore, cytotoxicity is induced, the cell cycle is arrested, and apoptosis is promoted.

The isolated terpineol decreases cell proliferation (Listiyana et al., 2023). *Artemisia absinthium* extract induces oral carcinoma cell cytotoxicity (Tsamesidis et al., 2024). Spilanthes paniculata flower essential oil extract inhibits OSCC proliferation and induces apoptosis (Satapathy et al., 2024). The isolated thymoquinone eliminates pre-malignant and malignant OSCC cells through cytotoxic mechanisms (Dagtas and Griffin, 2021). However, relatively few studies have investigated the anti-HNSCC effects of monoterpenoid compounds, and further research is needed (Figure 3) (Table 2).

**TABLE 2:** The anti-tumor effects and mechanisms of terpenoids and volatile oils against HNSCC.

Classification	Plant extracts	Mechanism of action	Tumor progression	Tumor	Ref
Monoterpenes	The isolated terpineol	NA	Decreases cell proliferation	OSCC	Listiyana et al. (2023)
	*Artemisia absinthium* extract	NA	Induces cytotoxicity in oral carcinoma cells	oral cancer	Tsamesidis et al. (2024)
	Spilanthes paniculata flower essential oil extract	NA	Inhibits OSCC proliferation and induces apoptosis	OSCC	Satapathy et al. (2024)
	The isolated thymoquinone	Cytotoxic mechanisms	Eliminates pre-malignant and malignant OSCC cells	OSCC	Dagtas and Griffin (2021)
Sesquiterpenes	The isolated megastigmane	NA	Exerts tumor-specific cytotoxicity	OSCC	Orabi et al. (2023)
	SRCR extract	NA	Induces apoptosis and inhibits migration	OSCC	Chang et al. (2023)
	A naturally isolated sesquiterpene lactone	NA	Induces apoptosis and inhibits migration in CAL-27 OSCC cells	OSCC	Shams et al. (2023)
	DRE	Inhibits PI3K/AKT and Ras/Raf/ERK signaling pathways and endogenous CBS/H2S axis	Inhibits growth, proliferation, migration and invasion of ESCC cells	ESCC	Duan et al. (2021)
Diterpene	The isolated CI-A	Oxidative stress and ERK activation	Suppresses OSCC proliferation and induces apoptosis	OSCC	Chuang et al. (2025)
	The isolated ginkgolide B	Knocks down PAER to inhibit ERK and AKT signaling pathways	Reverses cisplatin chemoresistance in OSCC	OSCC	Kaw et al. (2021)
	*Croton tonkinensis* extract (enriched in diterpenoids)	Inhibit PI3K/mTOR signaling	Suppress OSCC proliferation, apoptosis and autophagy, and enhance radiotherapy sensitivity	OSCC	Lee et al. (2024)
	The isolated triptolide	Suppresses PD-L1 expression in IFN-γ-rich OSCC microenvironments	Inhibits tumor growth and reverses immune evasion	OSCC	Kuo et al. (2021)
	The isolated RA	Inhibits AKT/mTOR/p70S6K pathway and activates JNK pathway	Triggers apoptosis	OSCC	Chen Z. et al. (2024)
	The isolated carnosol	Suppresses AKT/mTOR/LC3I/II pathway	Inhibits OSCC cells autophagy and decreases apoptosis	OSCC	Alaa El-Din et al. (2022)
	The isolated GLA	Amplifies ROS-driven ATF4/CHOP/CHAC1 cascade, triggers ER stress, redox imbalance, and apoptosis	Suppresses OSCC tumor growth	OSCC	Wang S. et al. (2022)
Triterpene	KFE	NA	Exhibits significant cytotoxic effects on OSCC cells and induces autophagy	OSCC	Kim et al. (2023)
	EEOS	NA	Exhibits various prooxidative and apoptotic effects	HNSCC	Utispan et al. (2023)
	Bacopa monnieri extract	Apoptosis	Inhibits OSCC proliferation	OSCC	Mishra et al. (2024)
	​	Blocks NLRP3 inflammasome via mitophagy	Suppresses immune evasion	OSCC	Mishra et al. (2024)
	The isolated actein	Activates FoxO1 tumor suppressor pathway	Inhibits OSCC proliferation	OSCC	Zhao et al. (2021)
​	The isolated cucurbitacin B	Inhibits PI3K/AKT signaling, induces G_2_ arrest and apoptosis	Suppresses OSCC proliferation and migration	OSCC	Yu et al. (2024)
	The isolated ailanthone	Inhibits PI3K/AKT signaling, downregulates CDK1/Cyclin B1, and induces G2/M arrest	Suppresses TSCC proliferation	TSCC	Wang S. et al. (2022)
	The isolated betulinic acid and koetjapic acid	Suppress AKT/mTOR, NF-κB, and JAK/STAT3 pathways	Inhibit OSCC survival, proliferation, invasion, angiogenesis, and metastasis, while simultaneously induce apoptosis	OSCC	Aswathy et al. (2021)
Others	*Juniperus indica* Bertol extract	NA	Inhibits ESCC growth and triggers cell cycle arrest and apoptosis	ESCC	Gao et al. (2025)
	The isolated fucoxanthin	Suppresses JAK/STAT3 pathway	Accelerates apoptosis	OSCC	Feng et al. (2023)

#### 2.2.2 Sesquiterpenes

The isolated megastigmane demonstrates tumor-specific cytotoxicity toward OSCC cells (Orabi et al., 2023). *Sparganii Rhizoma* and *Curcumae Rhizoma* (SRCR) extract induce apoptosis and inhibits migration in OSCC cells (Chang et al., 2023). A naturally isolated sesquiterpene lactone induces apoptosis and inhibits migration in CAL-27 OSCC cells (Shams et al., 2023). Dandelion root extract (DRE) suppresses esophageal squamous cell carcinoma (ESCC) cell growth, proliferation, migration, and invasion through inhibition of the PI3K/AKT, Ras/Raf/ERK, and endogenous CBS/H_2_S axis pathways (Duan et al., 2021). Research on the anti-HNSCC effects of sesquiterpenoids focuses on inducing apoptosis and inhibiting migration. Further studies on other aspects are still needed.

#### 2.2.3 Diterpenes

Diterpenoid compounds play a role in HNSCC, including by inhibiting cell proliferation and inducing apoptosis and autophagy, thereby suppressing the progression of HNSCC. The isolated (1R*, 12R*)-dolabella-4 (Hołota and Posmyk, 2025), 7,10-triene-3,13dione (CI-A) suppresses OSCC proliferation by inducing apoptosis via oxidative stress and ERK activation (Chuang et al., 2025). The isolated ginkgolide B reverses cisplatin resistance in OSCC by knocking down PAFR to inhibit ERK and AKT signaling (Kaw et al., 2021). *Croton tonkinensis* extract (enriched in diterpenoids) exerts anti-proliferative and radiosensitizing effects by blocking the PI3K/AKT/mTOR signaling axis and activating three death pathways: apoptosis, ferroptosis, and autophagy (Lee et al., 2024). The isolated triptolide inhibits tumor growth and reverses immune evasion through suppression of PD-L1 expression in IFN-γ-rich OSCC microenvironments (Kuo et al., 2021). The isolated Rhopaloic acid A(RA) triggers apoptosis in OSCC cells by inhibiting the AKT/mTOR/p70S6K pathway and activating the JNK pathway (Chen W.-F. et al., 2024). The isolated carnosol inhibits OSCC cell autophagy and decreases apoptosis through the suppression of the AKT/mTOR/LC3I/II pathway (Alaa El-Din et al., 2022). The isolated glaucocalyxin A (GLA) suppresses OSCC tumor growth through amplifying the ROS-driven ATF4/CHOP/CHAC1 cascade, triggering ER stress, redox imbalance, and apoptosis (Wang X. et al., 2022). In summary, diterpenoids can inhibit cell proliferation by inducing oxidative stress and ERK activation, by inhibiting the PI3K/mTOR signaling pathway, and by targeting the PAFR pathway. They can also induce apoptosis in OSCC cells and reverse immune evasion, thereby inhibiting the progression of HNSCC and demonstrating great potential as candidate drugs for OSCC treatment. However, most of these findings are based on *in vitro* cell experiments, and the *in vivo* efficacy, systemic toxicity, and crosstalk between various pathways still need to be further explored. This provides a direction for future research.

#### 2.2.4 Triterpenes

Triterpenoids are important drugs for treating HNSCC. They exert their anti-cancer effects by inhibiting proliferation and migration, inducing apoptosis, regulating autophagy, and suppressing immune evasion, thereby preventing the occurrence and development of HNSCC. Kochiae fructus extract (KFE) has significant cytotoxic effects on OSCC cells and induces autophagy (Kim et al., 2023). The ethanolic extract of *Ocimum sanctum* (EEOS) exhibits various prooxidative and apoptotic effects in HNSCC (Utispan et al., 2023). Bacopa monnieri extract inhibits OSCC proliferation through apoptosis and suppresses immune evasion by blocking the NLR family pyrin domain containing 3 (NLRP3) inflammasome via mitophagy (Mishra et al., 2024). The isolated actein inhibits OSCC proliferation through activating the FoxO1 tumor suppressor pathway (Zhao et al., 2021). The isolated cucurbitacin B suppresses OSCC proliferation and migration by inhibiting PI3K/AKT signaling and inducing G_2_ arrest and apoptosis (Yu et al., 2024). The isolated ailanthone suppresses TSCC proliferation through inhibition of PI3K/AKT signaling, downregulation of CDK1/Cyclin B1, and induction of G2/M arrest (Wang S. et al., 2022). The isolated betulinic acid and koetjapic acid inhibit OSCC survival, proliferation, invasion, angiogenesis, and metastasis by suppressing the activity of the AKT/mTOR, NF-κB, and JAK/STAT3 pathways while simultaneously inducing apoptosis (Aswathy et al., 2021). In summary, various natural products of triterpenoids, including those from the fruits of Coccinia indica and betulinic acid, have been proven to exert anti-cancer effects in OSCC through the induction of oxidative stress, DNA damage, and cell cycle arrest and the regulation of autophagy via PI3K/AKT, NF-κB, and FoxO1. However, research on their mechanism of action is still mostly limited to preclinical cell models, and further validation through *in vivo* experiments and clinical trials is lacking. The specific active ingredients, bioavailability, and potential toxicity of these compounds still require in-depth exploration.

#### 2.2.5 Other

In addition to the above-mentioned single terpene drugs, mixed terpene drugs can also have anti-HNSCC effects. *Juniperus indica* Bertol extract combined with 5-FU inhibits ESCC growth and triggers cell cycle arrest and apoptosis (Gao et al., 2025). The isolated fucoxanthin accelerates the apoptosis of OSCC cells by suppressing the JAK/STAT3 pathway (Feng et al., 2023).

### 2.3 Alkaloid

The isolated aporphine alkaloids exhibit anti-cancer activity in OSCC models (Machado et al., 2023). The isolated sanguinarine inhibits protective autophagy through inhibition of the PKM2/TFEB axis, thereby inhibiting the progression of OSCC (Peng et al., 2025).

### 2.4 Polysaccharides

The isolated ganoderma lucidum polysaccharides suppress cancer stemness and EMT (de Camargo et al., 2022). The isolated trehalose inhibits OSCC invasion through downregulation of the expression of the EMT transcription factor Slug (Shin et al., 2021). The isolated fucoidan reverses cisplatin resistance and increases OSCC apoptosis although it inhibits the PI3K/AKT pathway (Yang et al., 2023). *Phellinus igniarius* extract inhibits OSCC proliferation, migration, invasion, and overall tumor development through upregulating miR-655-3p expression (Li et al., 2023) (Table 3).

**TABLE 3:** The anti-tumor effects and mechanisms of polysaccharides against HNSCC.

Plant extracts	Mechanism of action	Tumor progression	Tumor	Ref
The isolated ganoderma lucidum polysaccharides	NA	Suppresses cancer stemness and EMT	TSCC	de Camargo et al. (2022)
The isolated trehalose	Downregulates EMT transcription factor Slug	Inhibits OSCC invasion	OSCC	Shin et al. (2021)
The isolated fucoidan	Inhibits PI3K/AKT pathway	Reverses cisplatin resistance and enhance OSCC cells apoptosis	OSCC	Yang et al. (2023)
*Phellinus igniarius* extract	Upregulates tumor-suppressive miR-655-3p	Inhibits OSCC proliferation, migration, invasion, and overall tumor development	OSCC	Li et al. (2023)

These studies have revealed the specific effects of polysaccharides on OSCC and some of their molecular mechanisms. However, the existing evidence is still limited to the preclinical stage, and the mechanisms focused on by each study are relatively isolated and lack systematicity. Future research needs to further integrate these scattered pathways and conduct verification in models closer to the human body to assess their true clinical translational value.

### 2.5 Phenylpropyl compounds

The isolated safrole inhibits proliferation and angiogenesis in OSCC (Kaur et al., 2023). The isolated sesamol induces OSCC cytotoxicity through mitochondrial apoptosis in SCC-25 cells (Ezhilarasan et al., 2021). *Cinnamomum zeylanicum* extract (CZE) and its bioactive compound cinnamaldehyde (CIN) inhibit OSCC proliferation and growth and induce apoptosis, cell cycle arrest, and autophagy via various cellular pathways (Aggarwal et al., 2022).

### 2.6 Other plant extracts

In addition to flavonoids, terpenoids and volatile oils, some macromolecular and small molecular drugs or metabolites, as well as proteins, can also exert certain anti-HNSCC effects. Goniothalamus umbrosus hexane extract (GUHE) is selectively cytotoxic to OSCC cells (Aziz et al., 2025). The isolated viscum album lectin induces OSCC apoptosis and disrupts cell cycle progression (Hong and Lyu, 2025). The isolated annonaceous acetogenins induce apoptosis in OSCC-15 cells by activating p53, upregulating BAX, and downregulating Bcl-2 (Mary et al., 2023). The isolated hexadecanoic acid inhibits OSCC cell growth and induces apoptosis via caspase-3 activation (Budi et al., 2022). The isolated Rhinacanthin-C (Rh-C) results in S-phase arrest and apoptosis in OSCC cells through the suppression of AKT survival signaling and the activation of p38 stress pathways (Klaophimai et al., 2023). The isolated dioscin inhibits OSCC proliferation by activating the RASSF1A/MST2/YAP axis, induces cell cycle arrest and promotes apoptosis through YAP phosphorylation and transcriptional inactivation (Tian et al., 2021). The isolated cannabidiol exerts cytotoxic and antimetastatic effects on OSCC cells by reducing survival and suppressing migration (Huang et al., 2022). A variety of natural extracts and their active components (such as GUHE, exadecenoic acid, hemagglutinin from Sophora flavescens, CBD, etc.) can effectively inhibit the growth of OSCC through promoting apoptosis, inducing cell cycle arrest, and suppressing cell migration through multiple pathways; their molecular mechanism involves the regulation of key signaling pathways such as caspase-3, AKT/p38, p53/BAX/Bcl-2, and RASSF1A/MST2/YAP (Table 4).

**TABLE 4:** The anti-tumor effects and mechanisms of other plant extracts against HNSCC.

Plant extracts	Mechanism of action	Tumor progression	Tumor	Ref
GUHE	NA	Exerts selective cytotoxicity against OSCC cells	OSCC	Aziz et al. (2025)
The isolated viscum album lectin	NA	Induces OSCC cells apoptosis and disrupts cell cycle progression	OSCC	Hong and Lyu (2025)
The isolated annonaceous acetogenins	Activates p53, upregulating BAX, and downregulating Bcl-2	Induces apoptosis in OSCC-15 cells	OSCC	Mary et al. (2023)
The isolated hexadecanoic acid	Via caspase-3 activation	Inhibits OSCC cells growth and induces apoptosis	OSCC	Budi et al. (2022)
The isolated Rh-C	Suppresses AKT survival signaling and activates p38 stress pathways	Results in S-phase arrest and apoptosis	OSCC	Klaophimai et al. (2023)
The isolated dioscin	Activates RASSF1A/MST2/YAP axis	Inhibits OSCC proliferation	OSCC	Tian et al. (2021)
​	YAP phosphorylation and transcriptional inactivation	Induces cell cycle arrest and apoptosis	OSCC	Tian et al. (2021)
The isolated cannabidiol	Reduces survival and suppressing migration	Exerts cytotoxic and antimetastatic effects against OSCC cells	OSCC	Huang et al. (2022)

### 2.7 Mixture

In addition to the individual extracts, the mixture also has the same anti-HNSCC effect. The ethanol extract of *Ocimum sanctum* (Holy Basil) demonstrates anti-cancer effects against OSCC cells (Luke et al., 2021). Derris scandens and Elephantopus scaber extracts induce apoptosis in OSCC cells (Leenutaphong et al., 2022). *Rosa foetida* essential oil extract possesses anti-cancer activity and strong antioxidant activity (Rajabi-Moghadam et al., 2025). Osbeckia octandra leaf extract inhibits OSCC proliferation by inducing G_1_ cell cycle arrest (Kim J. Y. et al., 2022). Areca nut extract inhibits OSCC proliferation through promoting cell cycle arrest and potentially triggering apoptosis (Sari et al., 2021). Triphala extract induces apoptosis through the inhibition of PI3K/AKT signaling in OSCC and inhibits proliferation, migration, and invasion (Hu S. et al., 2024). *Oroxylum indicum* stem bark extract suppresses OSCC progression by inhibiting the EGFR/PI3K/AKT pathway (Parvin et al., 2023). *Ocimum basilicum* extract exerts cytotoxicity to oral cancer cells by inhibiting cell cycle progression and inflammatory mediators (Li M. et al., 2024). Rudraksha extract mediated silver nanoparticles (REMAG) demonstrates significant cytotoxic effects against various cancer cell lines, suggesting that these nanoparticles may inhibit tumor growth and induce apoptosis (Sagar et al., 2025). The bioactive compounds present in Peganum harmala, such as harmaline and harmine, which are believed to inhibit cancer cell proliferation and induce apoptosis (Muddather et al., 2025). Caulerpa lentillifera extract demonstrates significant free radical scavenging ability, which may contribute to its protective effects against oxidative stress in cancer cells (Manmuan et al., 2025). Aloe vera extract has been shown to have active chemicals with anticancer effects, such as aloin and aloe-emodin. These substances may have anti-inflammatory, apoptotic, and cell proliferation-inhibiting properties. Additionally, by promoting the synthesis of cytokines, aloe vera can strengthen the immune system and assist the body fight off HPV infection and related cancers. Aloe vera may potentially work by modulating signaling pathways including the PI3K/AKT and MAPK pathway (Zhu et al., 2025). Anthemis maritima L. extract possesses considerable potential for developing therapeutic agents aimed at managing diseases characterized by unwanted cell migration, such as cancer (Mohammed et al., 2026). The isolated rhaponticin effectively inhibits cell proliferation, invasion, and migration, and reduces the expression of proteins in the IL6/STAT3 signaling pathway (Wei et al., 2025). The combination of garlic and fennel extracts achieves anti-cancer effects on OSCC by inhibiting cell proliferation and inducing apoptosis of cancer cells. Moreover, these natural extracts have a synergistic effect, enhancing their anti-cancer properties compared to single treatment (Prabhu et al., 2025). In summary, various plant extracts, through mechanisms such as inducing apoptosis, triggering cell cycle arrest, and inhibiting key oncogenic signaling pathways such as the PI3K/AKT and EGFR pathways, have been shown to have anti-proliferative, anti-migration and proapoptotic effects on OSCC *in vitro*. Moreover, some extracts can also inhibit inflammation-driven tumor occurrence by down-regulating inflammatory mediators, revealing the multi-target potential of plant extracts against OSCC. However, most of the related research is still at the *in vitro* stage, and *in vivo* efficacy, toxicity and bioavailability of plant extracts remain unknown; these key issues need to be addressed in future translational research (Table 5).

**TABLE 5:** The anti-tumor effects and mechanisms of mixture plant extracts against HNSCC.

Plant extracts	Mechanism of action	Tumor progression	Tumor	Ref
The ethanol extract of *Ocimum sanctum*	NA	demonstrates anti-cancer effect	OSCC	Luke et al. (2021)
Derris scandens and Elephantopus scaber extracts	NA	Induce apoptosis in OSCC cells	OSCC	Leenutaphong et al. (2022)
*Rosa foetida* essential oil extract	NA	Possesses anti-cancer activity and strong antioxidant activity	OSCC	Rajabi-Moghadam et al. (2025)
Osbeckia octandra leaf extract	Induces G_1_ cell cycle arrest	Inhibits OSCC proliferation	OSCC	Kim J. Y. et al. (2022)
Areca nut extract	Promotes cell cycle arrest and potentially triggers apoptosis	Inhibits OSCC proliferation	OSCC	Sari et al. (2021)
Triphala extract	Inhibits PI3K/AKT signaling	Induces apoptosis	OSCC	Hu S. et al. (2024)
​	​	Inhibits proliferation, migration, and invasion	OSCC	Hu S. et al. (2024)
*Oroxylum indicum* stem bark extract	Inhibits EGFR/PI3K/AKT pathway	Suppresses tumor progression	OSCC	Parvin et al. (2023)
*Ocimum basilicum* extract	Inhibiting cell cycle progression and inflammatory mediators	Exerts cytotoxicity on oral cancer cells	OSCC	Li M. et al. (2024)
REMAG	NA	Demonstrates significant cytotoxic effects, inhibits tumor growth and induces apoptosis	OSCC	Sagar et al. (2025)
The bioactive compounds present in Peganum harmala	NA	Inhibit cancer cell proliferation and induce apoptosis	OSCC	Muddather et al. (2025)
Caulerpa lentillifera extract	NA	Demonstrates significant free radical scavenging ability, which may contribute to its protective effects against oxidative stress in cancer cells	OSCC	Manmuan et al. (2025)
Aloe vera extract	NA	Has active chemicals with anticancer effects and has anti-inflammatory, apoptotic, and cell proliferation-inhibiting properties	OSCC	Zhu et al. (2025)
Anthemis maritima L. extract	NA	Possesses considerable potential for developing therapeutic agents aimed at managing diseases characterized by unwanted cell migration	OSCC	Mohammed et al. (2026)
The isolated rhaponticin	By the IL6/STAT3 signaling pathway	Inhibits cell proliferation, invasion, and migration, and reduces the expression of proteins	OSCC	Wei et al. (2025)
The combination of garlic and fennel extracts	NA	Inhibiting cell proliferation and inducing apoptosis of cancer cells	OSCC	Prabhu et al. (2025)

## 3 Mechanism of plant extracts regulating apoptosis/necroptosis/autophagy

**FIGURE 4:**
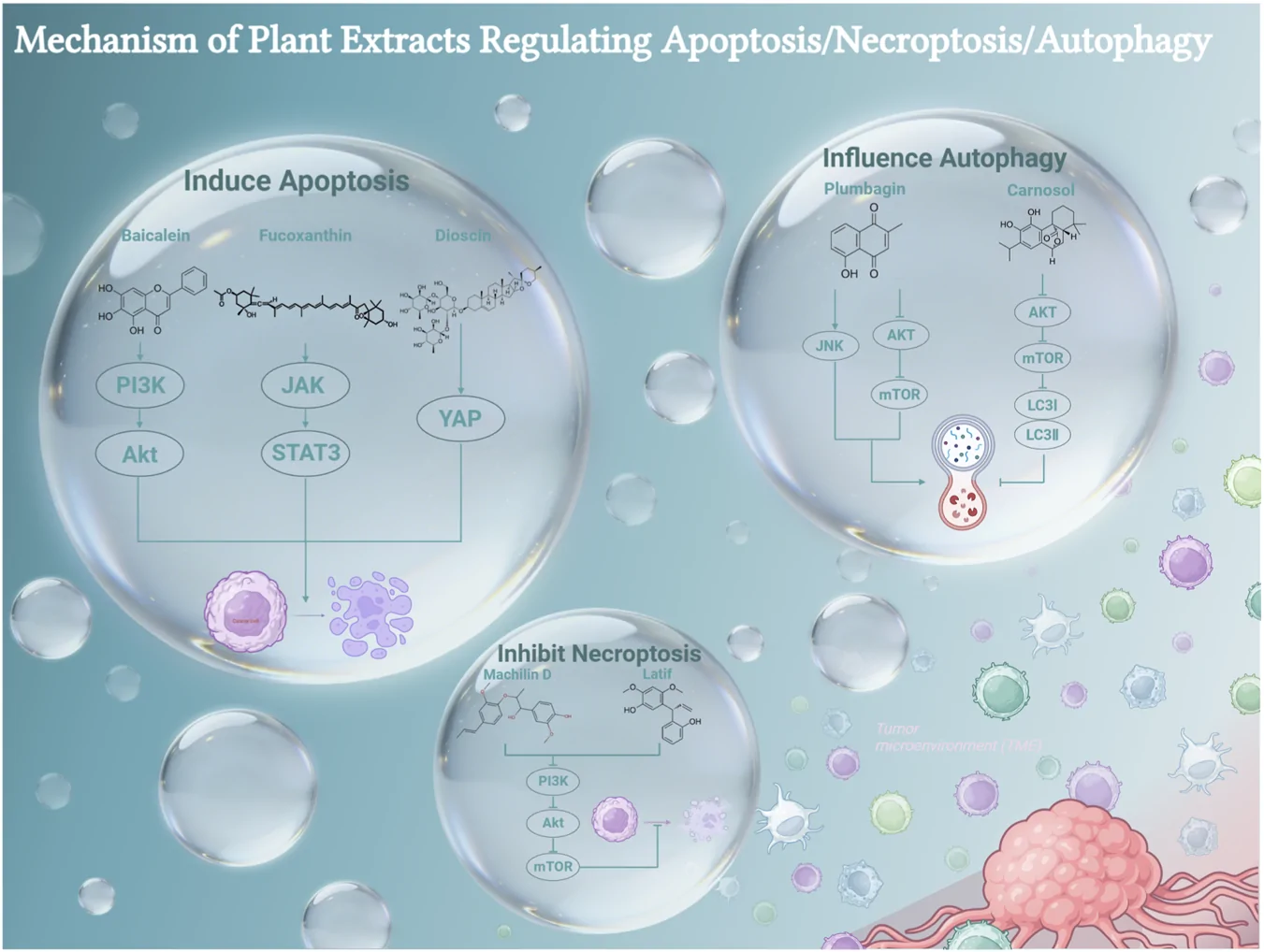
Mechanisms of plant extracts regulating apoptosis, necroptosis, and autophagy in HNSCC. Mechanisms by which plant extracts regulate apoptosis, necroptosis, and autophagy in HNSCC. Tumor cell apoptosis is induced via the PI3K/AKT, JAK/STAT3, and YAP pathways. Additionally, autophagy is either induced via the JNK and AKT/mTOR pathways or inhibited through the AKT/mTOR/LC3-I/LC3-II axis. Furthermore, necroptosis is inhibited through the PI3K/AKT/mTOR pathway.

Various plant extracts exert anti-HNSCC effects by regulating cell death differently. Among them, several plant extracts induce apoptosis in cancer cells through suppression of PI3K-AKT signaling (the isolated baicalein), suppression of the JAK/STAT3 pathway (the isolated fucoxanthin) and activation of YAP (the isolated dioscin) (Hou et al., 2021; Feng et al., 2023; Tian et al., 2021). Some plant extracts inhibit necroptosis through the inhibition of the PI3K/AKT/mTOR/p70S6K signaling pathway (the isolated latif and machilin D) and MAPK (the isolated machilin D) (Desai et al., 2025; Devi et al., 2025), and they also induce apoptosis. In terms of autophagy regulation, the isolated PLB extracts triggers apoptosis and autophagy by activating JNK and inhibiting the AKT/mTOR pathway, diterpenoids from *croton tonkinensis* promote autophagy through inhibiting PI3K/mTOR signaling, but the isolated carnosol and latif inhibit protective autophagy through suppressing the AKT/mTOR/LC3I/II pathway (Yun et al., 2022; Xue et al., 2021; Lee et al., 2024; Alaa El-Din et al., 2022). Notably, apoptosis and necropoptosis have a cross and balanced relationship on signaling pathways. By synergistically regulating these pathways and their related autophagic processes, plant extracts form a multi-targeted cell death regulatory network, providing an important mechanism for developing plant-derived therapies against HNSCC (Figure 4).

## 4 Regulatory signaling pathways

### 4.1 PI3K/AKT/mTOR

**FIGURE 5:**
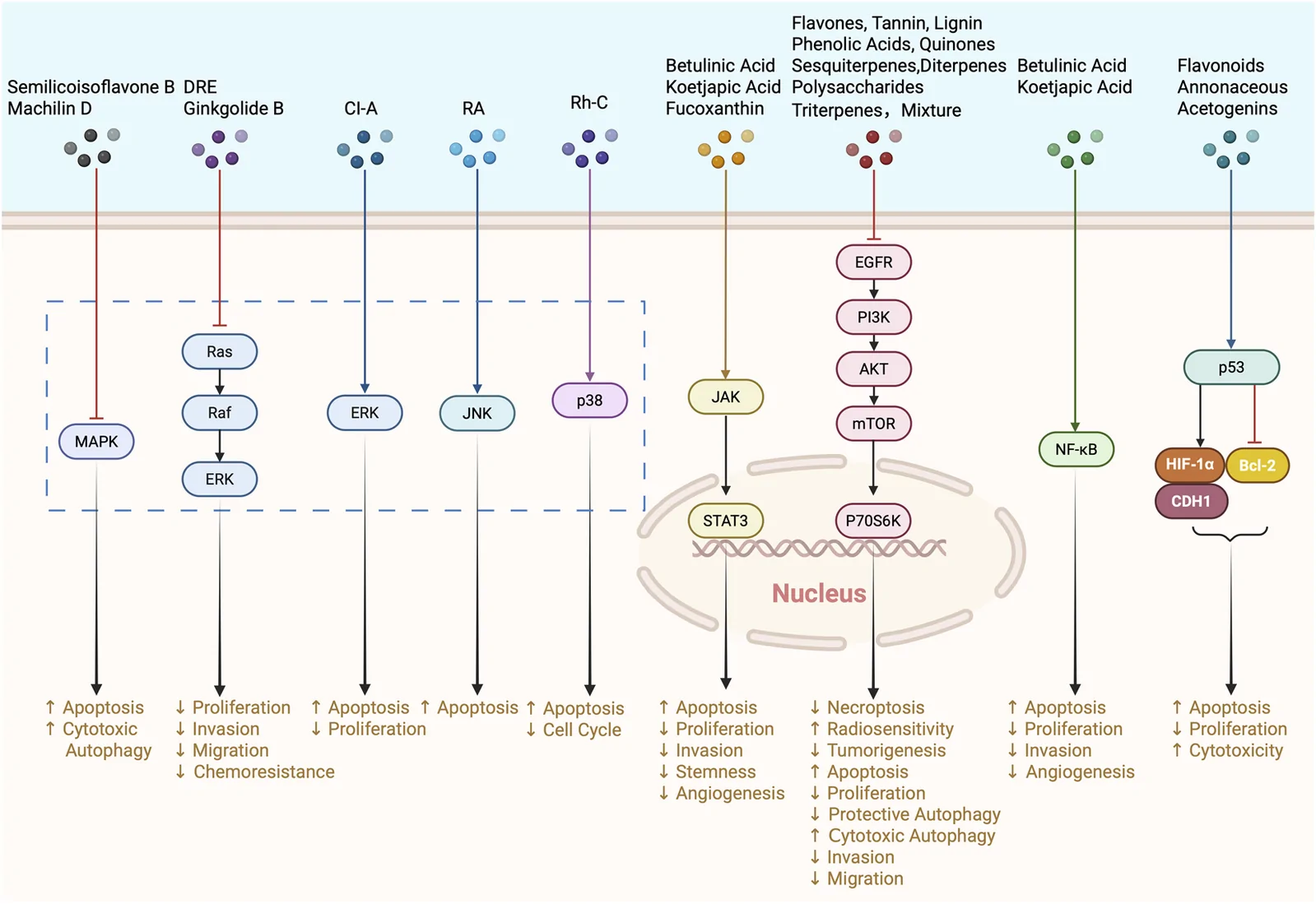
Signaling pathways implicated in anti-tumor activity of plant extracts against HNSCC. Multiple key signaling pathways, including MAPK, Ras/Raf/ERK, JNK, p38, JAK/STAT3, PI3K/AKT/mTOR, NF-κB, and p53, are targeted and regulated by plant extracts in HNSCC cells. Consequently, apoptosis, cytotoxic autophagy, cytotoxicity, and radiosensitivity are promoted, whereas cellular proliferation, invasion, migration, stemness, angiogenesis, tumorigenesis, protective autophagy, and chemoresistance are inhibited. Furthermore, cell cycle arrest is induced.

Plant extracts can regulate the PI3K pathway to inhibit proliferation, invasion and migration, promote apoptosis, regulate autophagy, inhibit necrotic apoptosis and enhance radiotherapy sensitivity, thereby interfering with the occurrence and development of tumors. Various plant extracts can inhibit the proliferation of OSCC cells through the PI3K/AKT/mTOR pathway, such as latif, baicalein, polymeric black tea polyphenols, GAME, DRE, and diterpenoids from *croton tonkinensis,* cucurbitacin B, ailanthone, Triphala betulinic acid and koetjapic acid (Yun et al., 2022; Hou et al., 2021; Nimbalkar et al., 2022; Nakamura et al., 2021; Duan et al., 2021; Lee et al., 2024; Yu et al., 2024; Wang S. et al., 2022; Aswathy et al., 2021; Hu S. et al., 2024). Various plant extracts can also promote the apoptosis of OSCC, such as latif, machilin D, PLB, RA, carnosol, cucurbitacin B, betulinic acid, fucoidan triphala, among other (Yun et al., 2022; Hou et al., 2021; Nimbalkar et al., 2022; Yun et al., 2023; Xue et al., 2021; Lee et al., 2024; Chen W.-F. et al., 2024; Alaa El-Din et al., 2022; Yu et al., 2024; Aswathy et al., 2021; Yang et al., 2023; Hu S. et al., 2024). Plant extracts have a dual effect on autophagy in cells through the PI3K/AKT/mTOR pathway. Some plant extracts inhibit protective autophagy, such as latif and carnosol (Yun et al., 2022; Alaa El-Din et al., 2022). Other plant extracts promote autophagy in OSCC, such as machilin D, diterpenoids from *croton tonkinensis*, and DRE (Yun et al., 2023; Duan et al., 2021; Lee et al., 2024). In addition, various plant extracts, such as baicalein, machilin D, cucurbitacin B, triphala, betulinic acid and koetjapic acid, can also inhibit the invasion and metastasis of OSCC through the PI3K/AKT/mTOR pathway (Hou et al., 2021; Yun et al., 2023; Yu et al., 2024; Aswathy et al., 2021; Hu S. et al., 2024). Latif and machilin D inhibit the necroptosis of OSCC cells by suppressing the activity of the PI3K/AKT/mTOR/p70S6K pathway (Yun et al., 2022; Yun et al., 2023). Diterpenoids from *croton tonkinensis* increase the sensitivity of OSCC to radiotherapy by inhibiting PI3K/mTOR signaling (Lee et al., 2024). Polyphenols from polymeric black tea and *oroxylum indicum* stem bark extract prevent OSCC occurrence and inhibit its progression through the suppression of EGFR/PI3K/AKT/mTOR signaling (Nimbalkar et al., 2022; Parvin et al., 2023). DRE inhibits the growth, proliferation, migration and invasion of ESCC cells through the inhibition of PI3K/AKT signaling (Duan et al., 2021) (Figure 5).

### 4.2 MAPK

The MAPK pathway is a hub family that transmits signals through a conserved kinase cascade. The three core branches downstream of ERK, JNK and p38 respond to growth factors and stress signals, precisely regulating key biological processes such as cell proliferation, differentiation, apoptosis and inflammation. Various plant extracts, such as SFBand machilin D, can induce autophagy and apoptosis in HNSCC cells through the downregulation of MAPK signaling (Hsieh et al., 2023; Yun et al., 2023).

#### 4.2.1 ERK

DRE inhibits the growth, proliferation, migration and invasion of ESCC cells by inhibiting Ras/Raf/ERK signaling (Duan et al., 2021). CI-A suppresses OSCC proliferation and induces apoptosis via oxidative stress and ERK activation (Chuang et al., 2025). Ginkgolide B reverses cisplatin chemoresistance in OSCC by knocking down PAFR to inhibit ERK signaling (Kaw et al., 2021).

#### 4.2.2 JNK

RA triggers apoptosis in OSCC cells by activating the JNK pathway (Chen W.-F. et al., 2024).

#### 4.2.3 p38

Rh-C causes S-phase arrest and apoptosis in OSCC cells through the activation of p38 stress pathways (Klaophimai et al., 2023).

### 4.3 JAK/STAT3

Betulinic acid and koetjapic acid inhibit OSCC survival, proliferation, invasion, angiogenesis, and metastasis by suppressing the activity of the JAK/STAT3 pathway while simultaneously inducing apoptosis (Aswathy et al., 2021). Fucoxanthin inhibits cell proliferation, stemness and migration while accelerating the apoptosis of OSCC cells through the inhibition of the JAK/STAT3 pathway (Feng et al., 2023).

### 4.4 p53 pathway

Flavonoids from PME inhibit OSCC proliferation and induce cytotoxicity through cell cycle arrest and gene regulation of p53, HIF one alpha, and CDH1 (Fonseca-Benitez et al., 2022). Annonaceous acetogenins induce apoptosis in OSCC by activating p53, upregulating BAX, and downregulating Bcl-2 (Mary et al., 2023).

### 4.5 NF-κB

Betulinic acid and koetjapic acid inhibit OSCC survival, proliferation, invasion, angiogenesis, and metastasis by suppressing NF-κB signaling and simultaneously inducing apoptosis (Aswathy et al., 2021).

### 4.6 Hippo

The dioscin axis inhibits OSCC proliferation through the activation of RASSF1A/MST2/YAP and induces cell cycle arrest and apoptosis through YAP phosphorylation and transcriptional inactivation (Tian et al., 2021).

## 5 Clinical translation

The clinical translation of plant extracts in HNSCC faces systematic challenges ranging from pharmacological limitations to shortcomings in the industrial supply chain. At the pharmacokinetic level, the coexistence of multiple components in extracts, poor water solubility of most active molecules, low intestinal absorption, and significant first-pass metabolism severely limits the oral bioavailability of key compounds, constituting the primary bottleneck for translation (Parvin et al., 2025; Pingping et al., 2025). To overcome this limitation, nanoformulation strategies (such as liposomes, polymeric micelles, solid lipid nanoparticles, and inorganic nanocarriers) have been highly anticipated. These nanoformulations can enhance solubility, prolong release, and achieve passive tumor targeting via the enhanced permeability and retention effect or active targeting through ligand modification, thereby partially overcoming pharmacokinetic disadvantages (Kaur et al., 2026). However, inherent issues such as low drug loading capacity, poor reproducibility in large-scale manufacturing, and potential immunotoxicity of nanocarriers also require careful consideration (Patwardhan et al., 2025).

Safety evaluation is non-negotiable. Studies on acute and chronic toxicity, genotoxicity, and interactions with radiotherapy and chemotherapy must be conducted in strict adherence to GLP standards (Duan et al., 2025; Abdollahi et al., 2026). Particular caution is needed regarding radiosensitization effects of plant constituents or their interference with drug-metabolizing enzymes. At the regulatory level, trial design must be purposefully aligned with the FDA’s Guidance on Botanical Drug Development or the EMA’s registration framework for traditional herbal medicinal products (Chen et al., 2025). This means treating the extract as a complex mixture, clearly delineating its role as adjuvant or alternative therapy, and underpinning that role with solid preclinical evidence. As the vital link between laboratory research and industrialization, quality control must rely on high-resolution mass spectrometry to establish multi-component chemical fingerprint profiles and implement multi-parameter quantitative analysis, ensuring end-to-end control from raw material origin and harvest time to manufacturing processes to guarantee batch consistency (Kumar et al., 2025; Zha et al., 2025). Commercialization challenges include supply volatility in sustainably sourced medicinal botanicals, the inherent scale-up complexity of multi-component manufacturing, and a void of market exclusivity arising from intrinsically weak patent protection, collectively eroding the industry’s incentive to drive clinical translation (Takubessi et al., 2025).

A number of phytochemicals have progressed beyond preclinical evaluation and entered clinical trials for HNSCC, thereby offering initial clinical evidence to guide translational research (Table 6). However, most clinical trials involving plant extracts for HNSCC remain single-center, small-sample exploratory studies, primarily focusing on short-term safety and surrogate endpoints, resulting in an extreme scarcity of high-level evidence-based data (Santiago et al., 2026; Ramsridhar et al., 2025). Future efforts urgently need to conduct multicenter, randomized, double-blind controlled trials integrating plant extracts with modern therapeutic approaches, incorporating pharmacokinetic biomarker monitoring and patient stratification, to truly pave the way for their entry into clinical practice.

**TABLE 6:** Phytochemical formulations in clinical trials.

Plant chemical preparations	Plant origin	Clinical trial stage	Main purpose	Source
Curcumin	Curcuma longa	Phase II/III	Prevention of second primary tumors after treatment for HNSCC	CTRI/2018/03/012274
Curcumin	Curcuma longa	Phase III	Curcumin mouthwash for preventing and alleviating oral mucositis caused by concurrent radiotherapy and chemotherapy for locally advanced HNSCC	TCTR20200820002
Quercetin	Various plants (such as onions, apples, etc.)	Phase II	Combination of Tislelizumab and Dasatinib for neoadjuvant treatment of resectable HNSCC (COIS-01)	NCT05724329
Polyphenon E	Camellia sinensis	Phase I	Combination of erlotinib for chemoprevention of precancerous lesions in the head and neck region	NCT01116336
Black raspberry extract	Rubus occidentalis	Phase 0/Early Phase I	A study on the “window of opportunity” from the time of HNSCC diagnosis to the time of surgery, evaluating changes in biomarkers	NCT06086925
APG-157	Plant-based pharmaceutical preparations of curcumin analogues	Phase II	Neoadjuvant/induction therapy for newly diagnosed locally advanced oral/opharyngeal head and neck squamous cell carcinoma	NCT05312710

## 6 Conclusion

**FIGURE 6:**
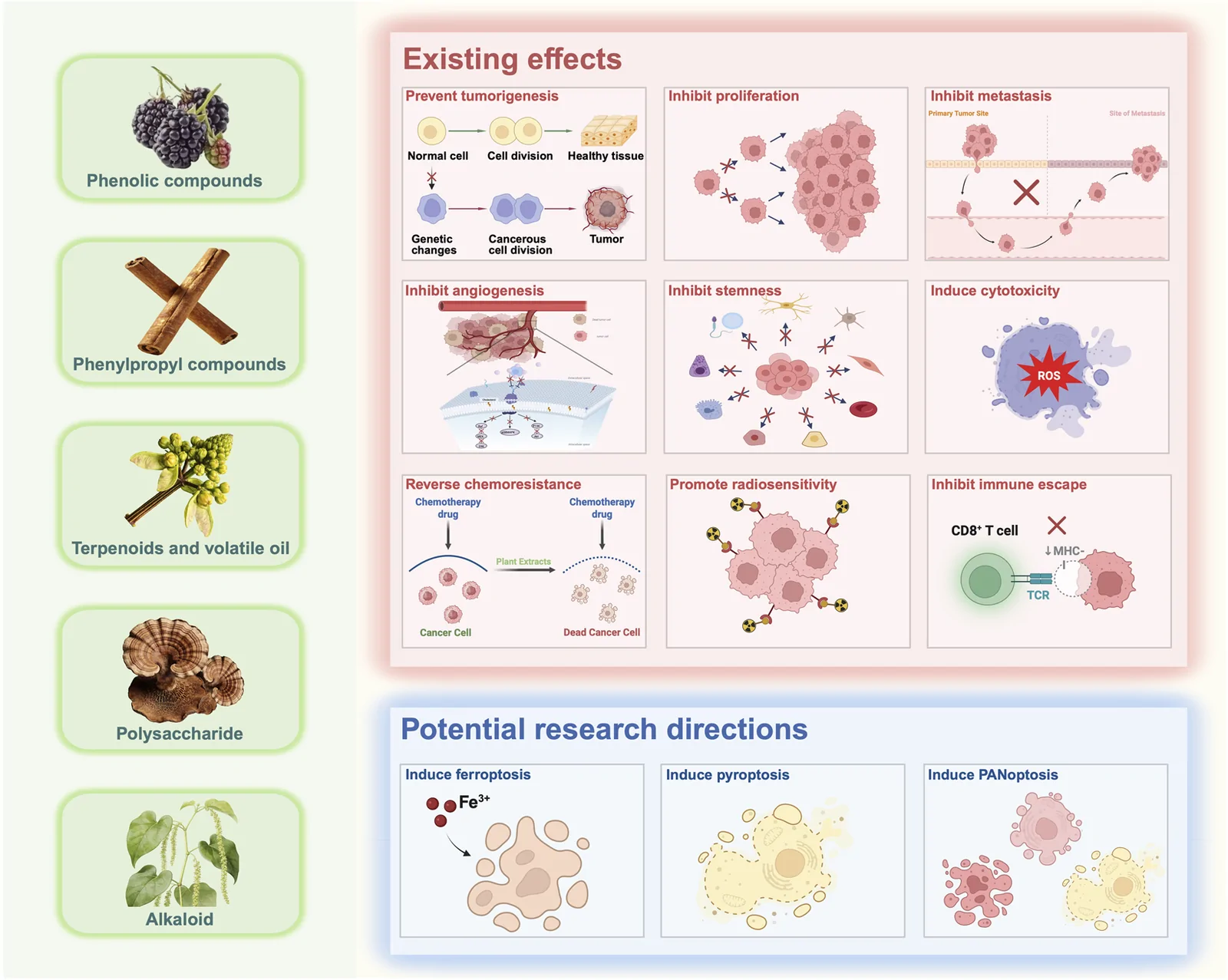
Existing anti-HNSCC effects of plant extracts and future potential research directions. Established mechanisms include prevention of tumorigenesis; inhibition of tumor cell proliferation, invasion, migration, metastasis, angiogenesis, stemness, chemoresistance, and immune escape; and promotion of radiosensitivity. Future perspectives include the exploration of plant extracts capable of inducing ferroptosis, pyroptosis, and PANoptosis in tumor cells.

**FIGURE 7:**
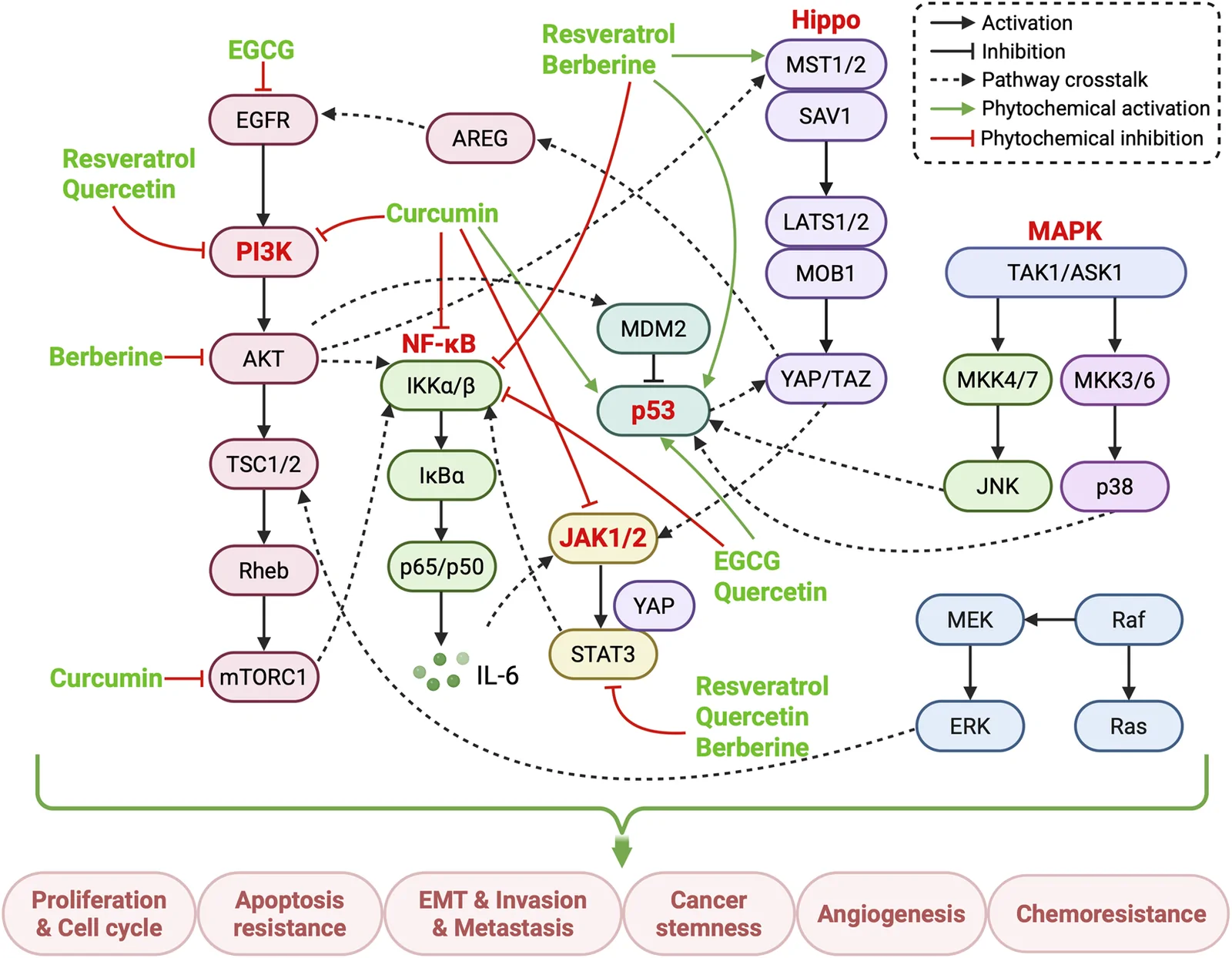
Multiple signaling networks modulated by plant extracts in HNSCC progression. There is some crosstalk among the PI3K/AKT/mTOR, MAPK, JAK/STAT3, NF-κB, Hippo, and p53 pathways in HNSCC. Plant extracts such as curcumin, EGCG, resveratrol, quercetin and berberine can inhibit HNSCC progression by targeting these pathways.

Several pure compounds, such as resveratrol, curcumin, quercetin, and anthocyanins, have demonstrated robust anti-HNSCC effects, including inducing apoptosis, inhibiting proliferation, blocking metastasis, and sensitizing cells to chemoresistance (Khan et al., 2022; Hazafa et al., 2020; Wan and Wang, 2025) (Figure 6). The PI3K/AKT/mTOR, MAPK, JAK/STAT3, NF-κB, Hippo, and p53 pathways constitute a highly interconnected signaling network rather than functioning as independent cascades during HNSCC progression. As a central signaling hub, AKT activates NF-κB signaling through IKK while promoting MDM2-mediated p53 degradation, thereby enhancing cell survival and suppressing apoptosis (Bai et al., 2009; Dan et al., 2008; Zhou et al., 2001; Ogawara et al., 2002). AKT also inhibits Hippo signaling, leading to YAP/TAZ nuclear accumulation and activation of pro-proliferative transcriptional programs (Gruber et al., 2016; Zanconato et al., 2016). Crosstalk between the MAPK and PI3K/AKT/mTOR pathways converges on the TSC2–mTORC1 axis, where ERK-mediated TSC2 phosphorylation sustains mTORC1 activation, whereas stress-activated JNK/p38 promotes p53-dependent cell-cycle arrest and apoptosis (Ma et al., 2005; Wu et al., 2004; Kim and Lozano, 2018). In parallel, NF-κB-induced IL-6 activates JAK/STAT3 signaling, while persistent STAT3 activity reinforces NF-κB transcription, establishing a positive feedback loop that drives inflammation, cancer stemness, immune evasion, and therapeutic resistance (Lee et al., 2009; Hu Z. et al., 2024; Johnson et al., 2018; Yu et al., 2014). Meanwhile, YAP/TAZ reactivates EGFR–PI3K/AKT and MAPK signaling through induction of EGFR ligands such as AREG and cooperates with STAT3 to amplify oncogenic transcription, whereas p53 counteracts YAP activity by inducing LATS2 and antagonizes NF-κB through competition for the transcriptional co-activators CBP/p300 (Gruber et al., 2016; Zanconato et al., 2016; Chen et al., 2022). Collectively, this extensive pathway crosstalk promotes HNSCC progression and therapeutic resistance while providing a mechanistic rationale for the multitarget activity of plant-derived compounds. By concurrently modulating multiple interconnected signaling pathways, these natural products may limit compensatory signaling and represent promising candidates for mechanism-based combination therapies in HNSCC (Figure 7).

The inconsistencies among studies on the anti-HNSCC effects of plant extracts mainly lie in differences in experimental models (e.g., 2D vs. 3D cultures, HPV-positive vs. HPV-negative HNSCC), extract standardization, and the comparative therapeutic efficacy of whole plant extracts versus isolated phytoconstituents. The vast majority of studies rely on 2D monolayer cultures and HPV-negative OSCC cell lines, with only a few employing more physiologically relevant 3D models and HPV-positive HNSCC cell lines (Fonseca-Benitez et al., 2022; Muddather et al., 2025; Zhu et al., 2025). This imbalance leads to inconsistent conclusions regarding both the recapitulation of the TME and the stratification by HPV status. A fundamental weakness in this field is the pervasive lack of extract standardization. Crude extracts are almost never accompanied by chemical fingerprinting or quantified active markers, and only a handful of isolated studies achieve partial qualitative and quantitative characterization (Kameyanda Poonacha et al., 2021; Nimbalkar et al., 2022). This deficit severely compromises inter-study comparability of pharmacodynamic data. In the direct efficacy comparisons with isolated phytoconstituents, whole-plant extracts exhibit superior antiproliferative or pro-apoptotic activities presumably via multicomponent synergy (Gusson-Zanetoni et al., 2022; Aggarwal et al., 2022). However, systematic comparisons of dose–response relationships and target engagement between these two forms remain lacking. Experimental model selection, chemical standardization, and pharmacological bridging of extracts and pure compounds remain critical unresolved bottlenecks, severely limiting the translation of experimental discoveries into clinical prevention or treatment strategies for HNSCC (Table 7).

**TABLE 7:** Clinical translational landscape of representative plant-derived agents for prevention and treatment of HNSCC.

Plant-derived agent	Primary molecular target(s)	Experimental evidence	Major antitumor mechanisms	Challenges for clinical translation	Clinical development stage in HNSCC	Ref.
Black raspberry gel	COX-2, iNOS (NOS2)	*In vivo*: DMBA-induced hamster buccal pouch carcinogenesis model Clinical: Phase II randomized placebo-controlled trial in patients with oral intraepithelial neoplasia (OIN)	Anti-inflammatory activity; inhibition of angiogenesis; induction of apoptosis; suppression of cell proliferation; promotion of cellular differentiation; prevention of progression from premalignant lesions to OSCC	Lack of formulation standardization and quality control; limited drug delivery strategies; tumor heterogeneity; insufficient precision medicine approaches; limited clinical evidence	Phase II clinical trial completed	Mallery et al. (2014)
Green tea extract	VEGF, Cyclin D1	*In vitro*: HNSCC cell lines Clinical: Phase II randomized placebo-controlled trial	Inhibition of VEGF-mediated angiogenesis; induction of apoptosis; induction of cell-cycle arrest	Limited formulation standardization; poor bioavailability and unfavorable pharmacokinetic properties; limited clinical evidence	Phase II randomized controlled trial (RCT)	Tsao et al. (2009)
Sulforaphane	NRF2, STAT3, SHH	*In vitro*: HNSCC cancer stem cell model *In vivo*: 4NQO-induced mouse model Clinical: Pharmacodynamic study in healthy volunteers	Activation of the NRF2/NQO1 antioxidant pathway; inhibition of STAT3 signaling; induction of caspase-dependent apoptosis; suppression of cancer stemness	Limited formulation standardization; poor bioavailability and pharmacokinetic limitations; limited clinical evidence	Early clinical translation	Bauman et al. (2016), Elkashty and Tran (2020)
Curcumin	SIRT1, IKKβ	*In vitro*: FaDu and CAL27 HNSCC cell lines *In vivo*: FaDu xenograft model Clinical: Pharmacodynamic study in HNSCC patients	Induction of caspase-dependent apoptosis; inhibition of angiogenesis; suppression of tumor invasion	Poor bioavailability and unfavorable pharmacokinetic properties; limited clinical evidence	Early clinical translation	Hu et al. (2015), Kim et al. (2011)
TriCurin	HPV16 E6/E7, STAT1	*In vitro*: HPV-positive HNSCC cell lines *In vivo*: HPV-positive HNSCC xenograft model	Induction of apoptosis; inhibition of colony formation and tumorsphere formation; remodeling of the tumor immune microenvironment	Lack of formulation standardization; limited drug delivery strategies; insufficient clinical evidence	Preclinical	Piao et al. (2017), Mukherjee et al. (2018)
Honokiol	EGFR	*In vitro*: SCC-1, SCC-5, OSC-19, FaDu and erlotinib-resistant HNSCC cell lines *In vivo*: SCC-1 and FaDu xenograft models	Induction of apoptosis; regulation of cell-cycle progression; enhancement of sensitivity to EGFR-targeted therapy	Poor bioavailability and unfavorable pharmacokinetic properties; limited clinical evidence	Preclinical	Leeman-Neill et al. (2010), Singh et al. (2015)
Resveratrol	REG III	*In vitro*/*In vivo*: HNSCC cell lines and xenograft models	Induction of DNA damage and apoptosis; suppression of tumor cell survival; enhancement of chemo- and radiosensitivity	Lack of formulation standardization; poor bioavailability and pharmacokinetic limitations; limited clinical evidence	Preclinical	Mikami et al. (2019), Masuelli et al. (2014)
Grape seed proanthocyanidins (GSPs)	EGFR, ERK1/2, NF-κB	*In vitro*: SCC1, SCC5, OSC19 and FaDu HNSCC cell lines *In vivo*: SCC1 xenograft mouse model	Induction of G1-phase cell-cycle arrest; induction of apoptosis	Lack of formulation standardization; poor bioavailability and pharmacokinetic limitations; limited clinical evidence	Preclinical	Prasad and Katiyar (2012)
Luteolin	p300	*In vitro*: CAL27 HNSCC cell line *In vivo*: CAL27 xenograft mouse model	Inhibition of histone acetylation; induction of cell-cycle arrest	Poor bioavailability and unfavorable pharmacokinetic properties; limited clinical evidence	Preclinical	Selvi et al. (2015)
Quercetin	MMP-2, MMP-9	*In vitro*: HSC-3, FaDu and other OSCC cell models	Suppression of cell migration, invasion and epithelial-mesenchymal transition; induction of growth arrest	Poor bioavailability and unfavorable pharmacokinetic properties; limited clinical evidence	Preclinical	Chan et al. (2016)
Apigenin	TNF receptor, DR5, Bcl-2, Bax	*In vitro*: SCC25 HNSCC cells treated alone or in combination with 5-fluorouracil (5-FU) or cisplatin	Induction of apoptosis; increased ROS generation; glutathione depletion; induction of G2/M-phase cell-cycle arrest	Poor bioavailability and unfavorable pharmacokinetic properties; limited clinical evidence	Preclinical	Chan et al. (2012)
Thymoquinone	N/A	*In vitro*: SCC25 and CAL27 HNSCC cell lines	Induction of apoptosis; enhancement of radiosensitivity	Poor bioavailability and unfavorable pharmacokinetic properties; limited clinical evidence	Preclinical	Kotowski et al. (2017)
Methanolic extract of *Withania somnifera*/Withaferin A	Bcl-2, Bax	*In vitro*: Human HNSCC cell lines	Induction of mitochondria-mediated apoptosis; promotion of cytochrome c release	Lack of formulation standardization; poor bioavailability and pharmacokinetic limitations; limited clinical evidence	Preclinical	Lee et al. (2016)
Ethanolic extract of *Apis mellifera* propolis	MMP-2, MMP-9	*In vitro*: HN30/HN31 and HN4/HN12 HNSCC cell lines	Induction of caspase-dependent apoptosis; suppression of tumor invasion	Lack of formulation standardization; poor bioavailability and pharmacokinetic limitations; safety concerns; limited clinical evidence	Preclinical	Niyomtham et al. (2021)

Emerging technologies such as nano-drug delivery systems, artificial intelligence, multi-omics, and personalized medicine can specifically address the current limitations of plant extract-based therapies and facilitate their clinical translation for HNSCC treatment. Nano-drug delivery systems systematically surmount the core bottlenecks (poor bioavailability, intrinsic instability, and off-target toxicity) of plant-derived bioactive compounds through solubilization, active targeting, and intelligent controlled release, thereby enabling selective and highly efficient accumulation of therapeutic payloads within HNSCC lesions (Gomerdinger et al., 2025). Artificial intelligence (AI)-driven optimization of extraction processes and intelligent quality control ensure batch-to-batch chemical consistency and bioequivalence of nanoformulations, fundamentally resolving the long-standing standardization hurdle that has constrained botanical drugs. Multi-omics technologies not only fortify quality control through modalities such as metabolomic fingerprinting, but also dissect tumor heterogeneity at depth, furnishing a precise molecular rationale for individualized treatment design (Miglino et al., 2025). The deep integration of AI with multi-omics comprehensively deciphers the multi-target synergistic regulatory mechanisms of plant-derived multi-components on HNSCC driver signaling pathways, immune microenvironment remodeling, and drug resistance networks, thereby accurately pinpointing pharmacodynamic biomarkers and optimal molecular combinations (Chang et al., 2025). Personalized medicine can precisely pair nano-engineered botanical preparations with molecularly stratified patient subgroups, and, by integrating multi-omics signatures with AI-driven therapeutic response models, truly deliver precision therapy that is high in efficacy, low in toxicity, and genuinely tailored to each patient (Reardon et al., 2026).

The current evidence indicates that Polyphenon E, curcumin, sulforaphane, and honokiol are the four plant components with the most promising clinical transformation prospects for HNSCC. Polyphenon E, as a standardized catechin extract that has undergone multiple phase I/II clinical studies, has clear safety, pharmacokinetic characteristics, and quality control processes, making it the most mature candidate drug at present (He et al., 2025). Curcumin has accumulated the most comprehensive basic research evidence through its multi-target regulation of multiple carcinogenic pathways and its synergistic effects with radiotherapy, chemotherapy, and immunotherapy (Pinto et al., 2026). Its bioavailability deficiency is being rapidly remedied by new nano-drug delivery systems. Sulforaphane demonstrates unique innovative mechanisms in overcoming drug resistance and blocking recurrence through Nrf2/Keap1, HDAC regulation, and inhibition of cancer stem cells and EMT (Adtani et al., 2025). It is regarded as an important molecular for the next-generation of precision treatment. Honokiol can efficiently inhibit pathways such as EGFR, PI3K/AKT/mTOR, and Hippo/YAP, and its good lipid solubility and tissue penetration ability can inhibit proliferation, metastasis, and angiogenesis. Combined with nanotechnology and immunotherapy strategies, it is expected to release greater clinical value (Dominiak et al., 2025). These four phytochemicals respectively demonstrate the most mature clinical evidence, the deepest basic research, the most original mechanism of action, and the most prominent emerging transformation potential, representing the most promising candidates for future clinical development.
